# Supporting follow-up care in colorectal cancer patients: first experiences with the Assessment of Burden of ColoRectal Cancer (ABCRC)-tool

**DOI:** 10.1007/s11764-024-01652-w

**Published:** 2024-08-16

**Authors:** Britt J. M. Thomassen, Anke H. C. Gielen, Jasmijn Baak, Meindert Sosef, Ester Ancion, Christel M. J. Gielen, Annerika H. M. Gidding-Slok, Stephanie O. Breukink, Merel L. Kimman

**Affiliations:** 1 Department of Surgery, Maastricht University Medical Centre+, Universiteitssingel 50 (UNS50) K5.442, 6229 ER Maastricht, The Netherlands; 2 Department of Gastroenterology and Hepatology, Maastricht University Medical Centre+, Maastricht, The Netherlands; 3 School of Nutrition and Translational Research in Metabolism (NUTRIM), Maastricht University, Maastricht, The Netherlands; 4 Faculty of Health, Medicine and Life Sciences, Maastricht University, Maastricht, The Netherlands; 5 Department of Surgery, Zuyderland Medical Center, Sittard-Geleen, The Netherlands; 6 Department of Family Medicine, Maastricht University, Maastricht, The Netherlands; 7 Care and Public Health Research Institute (CAPHRI), Maastricht University, Maastricht, The Netherlands; 8 School for Oncology and Developmental Biology (GROW), Maastricht University, Maastricht, The Netherlands; 9 Department of Clinical Epidemiology and Medical Technology Assessment (KEMTA), Maastricht University Medical Centre+, Maastricht, The Netherlands

**Keywords:** Patient-centred care, Follow-up, Patient-reported outcome measurement, Colorectal cancer, Burden of disease, Shared decision-making

## Abstract

**Purpose:**

The Assessment of Burden of ColoRectal Cancer (ABCRC)-tool is a unique tool that includes a PROM focused on health-related quality of life (HRQoL), functional outcomes and lifestyle assessment. Furthermore, it provides visualization of results and treatment advice. The tool aims to support follow-up consultations of colorectal cancer (CRC) patients. The purpose of this study was to evaluate the first experiences of patients and healthcare professionals (HCPs) with the ABCRC-tool.

**Methods:**

The ABCRC-tool was implemented in two Dutch hospitals and used by 25 patients and 5 HCPs during follow-up care. Consultations were audio-recorded and their content was analyzed independently by two researchers. Semi-structured interviews with patients and HCPs were conducted after the consultation. Interviews focused on the overall experience with the tool, ease of use, interpretation of the PROM and the visualized results and on the added value of the tool.

**Results:**

Audio recording revealed that multiple topics, relevant to patients, were discussed during consultations with the ABCRC-tool. Patients and HCPs appreciated the ABCRC-tool as it added structure to the consultation, was helpful in the preparation of consultations and provided useful and convenient treatment options. The tool was easy to use, and the visualization was clear and informative. HCPs suggested that the tool is likely to be most relevant for patients in the first year of follow-up.

**Conclusions:**

This study implies that the ABCRC-tool is of added value for patients and their HCPs. Future research should focus on the evaluation of broad implementation, across a wide range of CRC patients.

**Implications for Cancer Survivors:**

The ABCRC-tool is a valid tool to support CRC survivors and HCPs to monitor and visualize experienced burden of disease and lifestyle parameters in order to optimize personalized care.

## Introduction

Colorectal cancer (CRC) is the third most commonly diagnosed type of cancer worldwide [1]. As a result of national screening programmes, the early detection of CRC has significantly improved. This effect, combined with advancements in treatment options, has led to enhanced oncological outcomes, including improvements in disease-free and overall survival rates for CRC patients [2, 3]. As a result, the number of CRC survivors is growing. These patients may experience psychosocial and physical symptoms resulting from CRC treatment or the disease itself, such as anxiety, stress, fatigue, pain and defecation problems. These symptoms may lead to challenges in daily functioning, including social relationships and intimacy [4, 5].

Consequently, clinical guidelines acknowledge that follow-up care after treatment for CRC should increasingly focus on health-related quality of life (HRQoL) and functional outcomes [6]. Despite this recognition, follow-up consultations are still mostly provider focused and disease-driven, without systematic assessment of HRQoL and functional outcomes [7].

One way to incorporate the assessment and evaluation of HRQoL and functional outcomes in regular follow-up care is by using patient-reported outcome measures (PROMs). A review of the literature by Graupner et al. showed that the use of PROMs in daily cancer care resulted in improved HRQoL, symptoms, patient satisfaction and patient-physician communication, especially when feedback about the PROM results was provided to both patient and healthcare professional (HCP) [8]. With the aim of implementing PROMs in the CRC care pathway, te Boome et al. investigated the experienced disease burden of CRC patients and the most important issues relevant to be included in a PROM for CRC patients. Together with patients and HCPs, the ABCRC-tool was developed and validated [9, 10].

**Fig. 1:**
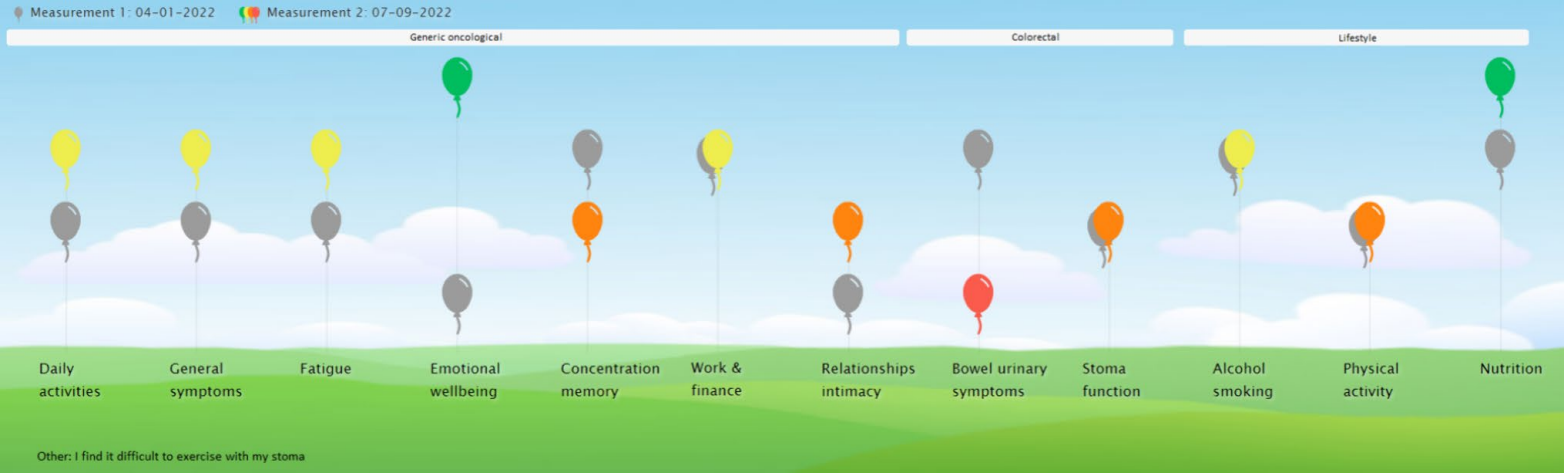
Balloon chart of the ABCRC-tool, with balloons indicating the patient’s outcome for the corresponding domain

The ABCRC-tool is unique as it contains a PROM, lifestyle assessment, visualization and treatment advice in one tool. The PROM consists of a generic module, assessing general oncological symptoms such as fatigue and physical functioning problems. It can be supplemented by one of four CRC disease specific modules, i.e. a module for colon cancer, rectum cancer, patients with a stoma and patients who participate in a watch-and-wait program [10]. The lifestyle assessment includes four items related to alcohol use, smoking, physical activity and nutrition. Patients can complete the PROM digitally at home, after which the results are immediately visualized in a balloon chart, together with treatment options that appear by hovering over the balloons (Fig. 1). The aim of this tool is to support follow-up consultations by promoting discussion of the issues that are most relevant to patients. This leads to more person-centred care and facilitates shared decision-making.

The aim of this study was to (1) evaluate the experiences of CRC patients and HCPs with the ABCRC-tool in daily oncological care and (2) describe lessons learned to optimize the further uptake of the ABCRC-tool in routine oncological care.

## Methods

### Design

The ABCRC-tool was implemented in two hospitals, one academic and one non-academic, in the southern region of the Netherlands. The study took place from July 2022 to January 2024. Recordings of consultations as well as semi-structured interviews were used to assess the content of the consultations and the experiences of patients and HCPs. Reporting of this study was done according to the Consolidated Criteria for Reporting Qualitative Research (COREQ).

### Participants

CRC patients > 18 years old and within the (5 years) postoperative follow-up phase of treatment were invited for participation by their nurse practitioner (NP) or physician assistant (PA). Patients could not participate if they were not proficient in written or spoken Dutch.

All patients provided written informed consent before participation, separately for audio recordings of the consultation and the interview. Their treating HCPs provided verbal consent for the audio recordings and for participation in an interview. We aimed to include at least ten patients in each hospital and continued until data saturation was reached.

### Data collection

#### Semi-structured interviews

Semi-structured interviews took place by telephone, Microsoft Teams or face-to-face. A semi-structured interview guide was developed with questions formulated as open as possible, allowing patients and HCP to elaborate on certain topics and address additional matters. As a final question, respondents were asked to grade their overall experience. The interview guide is provided in Appendixes 1 and 2. All interviews were conducted by BT, a female junior researcher who was trained in conducting interviews. Some interviews were attended by JB, a researcher in training. The interviewer had no pre-existing treatment relationship with the participants.

##### Patient interviews

Interviews with patients focused on all aspects of the practical use of the ABCRC-tool. First, patients were asked to elaborate on how they experienced completing the PROM of the ABCRC-tool at home, prior to the consultation. Thereafter, the interviews focused on the consultation in which the ABCRC-tool was used, e.g. if and how it was used, understanding of the balloon chart, and the usefulness and relevance of the treatment advices. Patients were given the opportunity to provide additional comments and suggestions regarding the ABCRC-tool and share their perspectives on its potential future use.

##### HCP interviews

Interviews with HCPs focused on their first impression of the ABCRC-tool, followed by their experiences regarding the practical use of the tool. HCPs were asked to reflect on the accessibility of the ABCRC-tool during the consultation (i.e. ease of use). HCPs were also asked to reflect on future implementation of the tool in daily practice.

#### Audio recordings of follow-up consultations

Consultations in which the ABCRC-tool was used were audio-recorded. A template was developed and used to document the type of consultation, type of HCP, follow-up phase, duration of the consultation and the topics discussed (Appendix 3).

### Data analysis

#### Semi-structured interviews

Both patient and HCP interviews were transcribed verbatim by JB, supported by Amberscript software for transcription. Transcripts were not returned to the participants. A thematic analysis approach with inductive coding was used. First, relevant sequences of the first five transcripts were labelled independently by BT and JB. These labels were compared and discrepancies were dissolved. Next, JB assigned preliminary codes to these transcripts, using ATLAS.ti 23.2.3. BT reviewed the coding procedure by checking coded transcripts at random. New codes and possible discrepancies were again discussed and dissolved, after which these codes were merged into broad themes by BT and JB.

#### Audio recording consultations

One researcher (either BT or JB) systematically examined the content of the recorded audio of the consultation, to complete the template and identify the topics discussed. Both researchers analyzed the first three consultations independently, and results were compared. No differences were identified; hence, for the remaining consultations, the analysis and completion of the template were performed by one researcher, with regular consultation with the second researcher to guarantee a unanimous approach.

## Results

A total of 25 patients and five HCPs participated in this study (Tables 1 and 2).

**Table 1:** Characteristics of patients

	Total *N* = 25
Sex, *N* (%)	
Male	15 (60)
Mean age (range), years	67.9 (38–84)
Module	
Colon with anastomosis	18
Colorectal with stoma	6
Rectum with anastomosis	1
Follow-up phase	
< 3 months	8
3–12 months	12
> 12 months	5

**Table 2:** Characteristics of HCPs

	Gender (M/F)	Hospital	Age (years)	Experience in (CRC) care (years)	ABCRC-tool usage (*N*)
Colorectal surgeon	M	Non-academic	55	18	2
Colorectal surgeon	F	Academic	50	24	8
Nurse practitioner	F	Academic	31	10	1
Nurse practitioner	F	Academic	52	32	25
Physician assistant	F	Non-academic	37	16	10

### Interviews

In total, 20 patients participated in an interview. The interviews were conducted on average 8 days (range 0–21 days) after the consultation and had a mean duration of 30 min (range 15 to 62 min). All HCPs were interviewed and these interviews lasted on average 22 min (range 16 to 31 min). No new information was yielded from the last three interviews, indicating that data saturation was reached.

#### Completing the PROM

Overall, patients considered the PROM to be relevant, clear, comprehensive and easy to complete. No problems were encountered with regard to the digital invite to access the PROM. Some patients perceived certain items as less relevant or preferred additional response options.

The questions are relevant, yes, complete as well from the mental state to the physical state. — P01

HCPs had similar experiences and mentioned that the open-ended question was not often used, suggesting that the items covered all relevant topics.

#### Preparation for the consultation

Patients indicated that they valued completing the PROM prior to their consultation, as it prompted them to contemplate and assess their current disease burden and wellbeing. Patients considered this helpful in preparation for the consultation. About 50% of the patients were able to see the visualization of the results, i.e. the balloon chart, at home. This was of great value to them and assisted in preparing the consultation.

As preparation for the consultation, it makes me think about how things are going. What are questions I would like to ask the physician? So it, it adds structure and I appreciate that. — P10

HCPs appreciated it when patients saw the balloon chart prior to the consultation, as it improved the interaction and communication during the consultation.

HCPs send the ABCRC-tool digitally to patients through the electronic patient file. HCPs mentioned that the method for sending out the PROM might require some initial exploration, but once known, it works properly.

#### General experience consultation

Overall, patients expressed positive attitudes towards the use of the ABCRC-tool. The tool facilitated structured discussions and the introduction of all relevant topics, including those not routinely addressed previously. This was attributed to patient’s unfamiliarity with the possibility to discuss these topics, as well as potential discomfort experienced by both HCPs and patients in initiating discussions on sensitive subjects, such as intimacy issues or sexual functioning. Furthermore, patients felt more involved and heard, as they were actively asked to indicate which topics they wanted to discuss.

I think it was a pleasant conversation, and I am convinced that, because structure was provided with the help of the list and the balloons with the topics underneath, much more was simply brought up, both from the doctor and from myself. — P10

Some HCPs noticed that the duration of the consultations increased with the use of the ABCRC-tool. However, according to HCPs, this might decrease again when experience is gained. At the same time, they felt that the quality of the consultation improved, because more emphasis was placed on the patients’ needs.

I think that in the end, the ABCRC-tool will save us time because after a while, patients will have less requests for help, receive better care and are happier. (…) isn’t that what we should aim for? — HCP02 (AH)

#### Balloon chart

The visualization of the results of the PROM in a balloon chart (Fig. 1) was considered a valuable addition to the consultations by both patients and HCPs. It provides a clear overview of the patient’s disease burden, and the balloons and colours were overall considered clear and self-explanatory. The option to see their previous results in grey appealed to patients, as this allowed them to see progress or worsening of outcomes over time. However, the meaning of the grey balloons was not immediately clear for some patients. A few patients did not find the balloons of added value. One patient deemed them childish and one patient experienced a negative association with the colour grey. For HCPs, the balloon chart provided a complete overview of the patients health status, and due to the colours, they could immediately detect domains that needed attention.

It is a tool to measure the burden of disease, and it shows: look, back then it was like this and now it is like this. Then you can see the progress or regression. — P04

#### Treatment advice

Most patients looked at the treatment advices and appreciated the help and advice to actively work on specific domains instead of only seeing a poor outcome. For some patients, the value was limited or, if outcomes were good, not applicable. One patient preferred not to see the balloons and treatment advices before the consultation, but rather discussed the outcomes with their HCP first. For HCPs, it was convenient to have an overview of potential treatment advices that could be discussed, especially for the less clinical topics, such as work and finance and lifestyle. For patients with a higher burden of disease, it was important to be able to access the results and treatment advices *after* the consultation as well.

What I do think, is that if you have people who are really struggling, they can definitely benefit a lot from this. When you fill in those specific complaints, you can then see below, hey, you can approach these caregivers or institutions, or do this and that — P11

I did take a look at them, but right now I actually don’t feel the need for it because, I’m currently in a relatively positive flow.* —* P05

#### Use of the ABCRC-tool in the future

The majority of patients would like to use the ABCRC-tool again in future consultations and find the tool of added value. Yet, some patients saw no benefits for themselves, as they had no or very few symptoms and a low disease burden. This was especially true for patients who were > 1 year post-treatment. HCPs also saw most value in the first year after treatment, when the patient’s disease burden is highest. They all stated that they wanted to continue using the ABCRC-tool in their follow-up care. It was expected that the tool would most likely be used by nurse practitioners or physician assistants, who are increasingly covering the follow-up care of CRC patients in The Netherlands.

Well, I would like to work with it and especially in the first year of the follow-up, because I believe that is the period in which most patients face difficulties and ask themselves: is it normal what I experience and how am I supposed to deal with it? — HCP03 (AH)

#### Overall experience of the ABCRC-tool

Patients graded the overall experience of the ABCRC-tool with an average of 8.0 on a scale of 1–10. Patients mainly based their grade on the fact that a variety of relevant topics were now discussed. The fact that they can use the PROM to prepare the consultation, see their outcomes in a balloon chart, in combination with the ability to keep track of changes over time, was considered of added value. Room for improvement was mainly seen in the option to visualize the balloon chart and treatment advice at home which did not function properly for some patients. Furthermore, some patients in their second and third year of follow-up, who were doing well, did not see much added value of the tool.

I think the tool is very user friendly. I like the way it guides you into a consultation, and also to recognize a certain trend over the long term. — P03

The participating HCPs graded the overall experience of the ABCRC-tool with average grade of 8.3. The HCPs considered the tool to be an improvement for CRC care. Nevertheless, the HCPs identified certain implementation barriers, particularly related to the duration of the consultation and technical challenges. Resolving these issues is deemed essential for the efficient integration of the tool into clinical practice.

I liked it because it is well structured (…) you prevent that either I will forget to ask about things, or that the patient does not dare to ask about it. — HCP01 (NAH)

It is of added value. It looks good, it is intuitive, it is easy, not too many questions, doesn’t take too long. It is of added value, both for the patient as for the healthcare professional. — HCP02 (AH)

### Audio recordings of follow-up consultations

Twenty-five consultations in which the ABCRC-tool was used were recorded. The mean duration of the consultations was 15 min (range 6 to 32 min). As this was the first time that these patients used the ABCRC-tool, a substantial part of the consultation involved explanation of the tool, which took some time. Table 3 provides an overview of the domains of the ABCRC-tool addressed during the consultations. It shows that nearly all domains of the ABCRC-tool were discussed in the majority of consultations. Domains such as “stoma” and “work and finance” are not applicable to all patients and were therefore less frequently discussed.

**Table 3:** Overview of the topics that were discussed during the recorded consultations

Domain	Topics	% (*N*)
General	Blood test	88 (22)
	CT scan	64 (16)
	Colonoscopy	64 (16)
Colorectal	Bowel, urinary symptoms	72 (18)
	Stoma function	32 (8)
Physical	Daily activities	60 (15)
	General symptoms	76 (19)
	Fatigue	64 (16)
Emotional	Emotional wellbeing	68 (17)
Cognitive	Concentration, memory	52 (13)
Social	Work, finance	40 (10)
	Relationships, intimacy	60 (15)
Lifestyle	Alcohol, smoking	64 (16)
	Physical activity	80 (20)
	Body weight, nutrition	44 (11)

Furthermore, these consultations provided quick insight into the practical use of the tool at that time. It became evident that both patients and HCPs had their own way of using the tool. Sometimes, all domains were briefly discussed, while in other consultations, the patient and HCP decided to focus on one or a few domains specifically. If any issues or ambiguities were identified within the audio recordings, researchers could act upon them immediately and provide additional instructions for future consultations.

## Discussion

The ABCRC-tool is developed to support personalized care and shared decision-making during follow-up care after CRC treatment. In this study, the first experiences of patients and HCPs regarding the use of the ABCRC-tool in daily care were explored by audio recording consultations and conducting interviews with patients and HCPs.

Overall, patients and HCPs concluded that use of the ABCRC-tool was of added value. The primary advantages of the tool include its utility in preparing consultations, providing structure to the discussions and introducing significant topics that might otherwise be overlooked. Additionally, the treatment advice offered is beneficial for HCPs and equips patients with effective tools to alleviate their disease burden independently, thereby contributing to self-management of patients. Our findings suggest that the tool might be of most value for patients relatively early in their follow-up.

The results of the interviews are in concordance with the information gained from the audio recordings of the consultations, where many domains were discussed in the majority of consultations, guided by the patient’s questions and needs at that time.

The audio recordings revealed that the mean duration of the consultations was 15 min. Sufficient time for the consultation was also mentioned as essential for successful implementation by Graupner et al. [11]. Since some time was spent on explanation of the tool, it is expected that once experience increases, the tool will be used more efficiently.

Ultimately, the instrument’s flexibility allows patients to use it according to their preferences, thereby maximizing the promotion of personalized care.

### In relation to other research

The ABCRC-tool has the potential to fulfil needs that were identified in earlier research. As CRC patients can experience a broad range of post-treatment symptoms and require focused and routine assessment of treatment consequences, clear referral pathways and adequate supportive care to address patient’s needs and improve (long-term) health outcomes is pivotal [12, 13]. This study has shown that the ABCRC-tool supports follow-up consultations by ensuring that treatment consequences relevant to patients are discussed, and therefore has the potential to positively impact patient outcomes. Moreover, the balloon chart and corresponding treatment advice have the potential to support self-management and remote care, which might be the preferred method of care for some patients [14]. As a substantial part follow-up consultations are already scheduled by phone nowadays, the ABCRC-tool might provide additional structure, depth and meaning to these consultations, especially when both the patient and HCP can access the balloon chart simultaneously.

### Strengths and limitations

This study has shed light on the content and course of consultations with the ABCRC-tool. Strengths of this study include the rigorous methodology with regard to the interviews, supplemented with the information gained from audio recordings to gain even better insight into the practical use of the tool.

Unfortunately, there is no data available that allows for direct comparison between consultations with and without the ABCRC-tool. Hence, it is not possible to conclude anything about changes in topics discussed or the durations of consultations as a direct result of the introduction of the ABCRC-tool. However, the main goal of this study was to gain insight into how the tool is used and what we can learn from this first implementation in two different hospitals. The ABCRC-tool was developed as a tool that patients can access from their home. Due to technical issues, unfortunately, not all patients in this study were able to see the balloon chart and corresponding treatment advice at home. Initially, this occurred because the balloon chart did not generate properly at specific devices, which was resolved during the study. Subsequently, it became apparent that it was not clear for some patients how to retrieve the balloon chart. Another limitation of this study is the lack of patients who are > 32 months in their follow-up. Moreover, this study reports the first experiences of two hospitals, of which one hospital was involved in the development of the tool.

### Future practice

To assist both HCPs and patients in navigating the digital and online features of the tool, pamphlets containing clear instructions were developed, and more attention will be allocated to training of HCPs. For further implementation of the tool, we suggest to introduce the tool to all patients during their first consultation post-surgery. Moreover, integrating the tool in a broader range of information technology systems and acknowledgement of the tool in national guidelines are important future steps to enhance overall implementation of the tool.

### Future research

Overall, this study has shown the potential added value of the ABCRC-tool for HCPs and CRC patients during follow-up and the feasibility of this tool in daily oncological care. The tool is ready for broader implementation and routine use. Future studies could investigate if use of the tool affects the quality of life, experienced quality of care or healthcare consumption of patients. It is envisaged that the lifestyle domain of the ABCRC-tool can be used to screen for CRC patients that could benefit from lifestyle interventions, as increasing evidence shows the beneficial effects of a healthy lifestyle on quality of life and disease-free and overall survival in CRC survivors [15–18]. Furthermore, the tool can easily be translated into other (oncological) disciplines in the future, partly because the foundation of the tool is a generic oncological module. In this study, patients also expressed their hope that other medical disciplines would implement a similar tool in the future. In addition, completed ABCRC-tools generate a wealth of data that could be used for future research into quality of life, lifestyle and disease burden of this patient group. Some patients also considered this important and even cited it as a reason to continue using the tool.

## Conclusion

The ABCRC-tool is of added value for patients who are in the follow-up phase of CRC treatment and their treating HCPs, especially in the first 1 or 2 years after surgical treatment. The tool is ready for wider implementation, to enhance the quality of CRC follow-up care with a personalized approach.

## Appendix 1. Interview guide—semi-structured interviews with patients (*originally in Dutch*)

- Study number
- Date of birth
- Sex
- Hospital in which treatment is given
- Timing of follow-up

### Introduction

The interviewer…

- briefly repeats the goal of the study;
- mentions their role within the study;
- checks once more whether the patient is willing to participate;
- checks whether the patient approves that the consultation is recorded for research purposes and emphasizes that the recording will be securely stored and results are used anonymously in a scientific publication;
- mentions the length of the interview;
- checks whether there are questions from the patient before starting the interview.

### Experiences regarding the questionnaire

- What was it like for you to fill in the questionnaire?
- How did you fill in the questionnaire, by phone or on a computer or laptop?
- What did you think of the questions?
- Were the questions formulated clearly?

If not, can you indicate what was unclear for you?

- What did you think of the way in which you received the questionnaire?
- What did you think of the visual appearance of the questionnaire? (format, readability, etc.)

### The consultation

- Can you tell me how you have experienced the consultation in which the ABCRC-tool was used?
- If you compare this consultation with previous consultations where you did not use the ABCRC-tool, are there differences notable?
  - If so, what kind of differences and how did you experience that?

### Balloon chart and treatment advices

- What did you think of the balloon chart?
- To what extent was the balloon chart helpful for you during the consultation? And after the consultation?
- Did you discuss (all of) the treatment advices with you doctor/nurse practitioner?

If so, what did you think of the treatment advices? Is it possible for you to act upon the given treatment advices?

If not, why not?

- What would you think of the possibility to have access to the balloon chart and treatment advices at home?
- Would you like to see the balloon chart and treatment advices previous to the consultation? Or only after the consultation?

### Experience ABCRC-tool

- How did you experience the use of the ABCRC-tool during the consultation?

If experience was positive; can you elaborate on what aspects went well/were pleasant?

If experience was negative; can you elaborate on what aspects did not go well/were unpleasant?

- Are there aspects, regarding the use of the ABCRC-tool, you did not like?

If so, what could be improved? Did you miss anything?

If not, what did you like about it?

### Future of ABCRC-tool

- The next time you will visit your healthcare professional, would you like to use the ABCRC-tool again or not? Why (not)?
- If you are going to use the ABCRC-tool again, is there anything you need?

### Grading the ABCRC-tool

- If you could grade the use of the ABCRC-tool between 1 and 10, what grade would you give and why?

### Household

- What does your household look like? (Did you involve someone (partner/family member) in filling in the questionnaire or did you discuss the ABCRC-tool with someone?)

### End

- Are there any more topics you would like to mention about the use of the ABCRC-tool/balloon chart, that we have not yet discussed?

The interviewer…

- checks whether the patient has remaining questions;
- informs the patient on how the study continues: the information that is gathered from the interviews will be used to improve daily care. In addition, a scientific article will be written about the ABCRC-tool. The information provided in the interviews will be processed anonymously;
- thanks the patient.

## Appendix 2. Interview guide—semi-structured interviews with HCPs (*originally in Dutch*)

- Study number
- Hospital
- Function

### Introduction

The interviewer…

- briefly repeats the goal of the study;
- mentions their role within the study;
- checks once more whether the patient is willing to participate;
- checks whether the patient approves that the consultation is recorded for research purposes and emphasizes that the recording will be securely stored and results are used anonymously in a scientific publication;
- mentions the length of the interview;
- checks whether there are questions before starting the interview.
- asks the healthcare professional:
  o function
  o in which hospital he/she is working and in which function
  o how long he/she is working there in its current function
  o how long he/she is working in health care
  o age
  o sex
  o how many time he/she has used the ABCRC-tool

### General first impression of ABCRC-tool

- What was it like for you to use the ABCRC-tool?
- What was your first impression of the ABCRC-tool?

### Practical experiences

- With how many patients have you used the ABCRC-tool?
- In what way have you used the ABCRC-tool?

Go through all the steps and ask how the healthcare professional experienced that part

- Have you seen the questions?

If so, what did you think of the questions?

What did you think of the way in which the questionnaires are send?

What did you think of the visual appearance of the questionnaire?

- Were there any unclarities regarding the use of the ABCRC-tool?

If so, what would you need to make it more clear?

What would you colleagues need?

- What was it like for you in general to use the ABCRC-tool during the consultation?
- Do you think that the ABCRC-tool is easy or difficult to use?

Why is that?

What would help you or your colleagues?

### EMR (Electronic Medical Record)

- Was the ABCRC-tool easy to find in the EMR or not?

If not, what would help you?

- Did it look well-organized?

If not, why not?

### Consultation

- Can you tell me how the consultation went in which the ABCRC-tool was used?
- If you compare this consultation to previous consultations where you did not use the ABCRC-tool, are there differences notable?

If so, what kind of differences and how did you experience that?

### Balloon chart and treatment advices

- What did you think of the balloon chart?
- Did you discuss (all of) the treatment advices with the patient?

If so, how did it go? How did the patient respond?

What did you think of the treatment advices?

What would you think of the possibility that the patient has access to the balloon chart and treatment advices at home?

### Use ABCRC-tool

- How did you experience the use of the ABCRC-tool during the consultation?

What would you say was challenging?

What would you (or your colleagues) need?

### Differences patients during consultations

- What was it like for you to use the ABCRC-tool with different patients?

Did you notice any differences between patients/consultations?

If so, what kind?

- What went well?
- What could still be improved?
- Did you miss anything?

### Grading the ABCRC-tool

- If you could grade the use of the ABCRC-tool between 1 and 10, what grade would you give and why?

### Future of ABCRC-tool

- To what extent would you like to keep working with the ABCRC-tool in daily care?

Why (not)?

What is your role herein?

### Implementation of ABCRC-tool

- Imagine, that your department will start working with the ABCRC-tool tomorrow.

What would you need?

What would your colleagues need? And why?

Would you run into anything?

How can we help you?

### End

- Are there any more topics you would like to discuss?

The interviewer…

- checks whether the healthcare professional has remaining questions;
- informs the healthcare professional on how the study continues: the information that is gathered from the interviews will be used to improve daily care. In addition, a scientific article will be written about the ABCRC-tool. The information provided in the interviews will be processed anonymously;
- thanks the healthcare professional.

## Appendix 3. Scoring sheet audio recordings of consultations (*originally in Dutch*)

**Table:** 

		Comments/quotes
Study number		
Assessor		
Date		
Duration of consultation (minutes)		
Type of consultation	Face-to-face	
	By phone	
Phase of follow-up	6 weeks	
	3 months	
	6 months	
	9 months	
	12 months	
	Other	
	Unknown	
Type of healthcare professional	Physician	
	Nurse practitioner	
	Physician assistant	
Discussed topics		
	Blood test	
	CT scan	
	Colonoscopy	
	Other, namely:	
*Colorectal*	Bowel, urinary symptoms	
	Stoma function	
*Physical*	Daily activities	
	General symptoms	
	Fatigue	
*Emotional*	Emotional wellbeing	
*Cognitive*	Concentration, memory	
*Social*	Work, finance	
	Relationships, intimacy	
*Lifestyle*	Alcohol, smoking	
	Physical activity	
	Body weight, nutrition	
*Other, namely:*		
